# Insight Into the Function of RIPK4 in Keratinocyte Differentiation and Carcinogenesis

**DOI:** 10.3389/fonc.2020.01562

**Published:** 2020-08-14

**Authors:** Jing Xu, Qichun Wei, Zhixing He

**Affiliations:** ^1^Department of Radiation Oncology, Key Laboratory of Cancer Prevention and Intervention, Ministry of Education, The Second Affiliated Hospital, Zhejiang University School of Medicine, Hangzhou, China; ^2^Institute of Basic Research in Clinical Medicine, College of Basic Medical Science, Zhejiang Chinese Medical University, Hangzhou, China

**Keywords:** RIPK4, signaling pathways, keratinocyte differentiation, therapeutic target, carcinogenesis

## Abstract

The receptor-interacting protein kinase 4 (RIPK4), a member of the RIPK family, was originally described as an interaction partner of protein kinase C (PKC) β and PKCδ. RIPK4 is identified as a key regulator of keratinocyte differentiation, cutaneous inflammation, and cutaneous wound repair. The mechanism by which RIPK4 integrates upstream signals to initiate specific responses remains elusive. Previous studies have indicated that RIPK4 can regulate several signaling pathways, including the NF-κB, Wnt/β-catenin, and RAF/MEK/ERK pathways. Furthermore, RIPK4-related biological signaling pathways interact with each other to form a complex network. Mounting evidence suggests that RIPK4 is aberrantly expressed in various kinds of cancers. In several types of squamous cell carcinoma (SCC), the mutations that drive aggressive SCC have been found in RIPK4. In addition, the function of RIPK4 in carcinogenesis is probably tissue-specific, since RIPK4 can play a dual role as both a tumor promoter and a tumor suppressor in different tumor types. Therefore, RIPK4 may represent as an independent prognostic factor and a promising novel therapeutic target, which can be used to identify the risks of patients and guide personalized treatments. In future, RIPK4-interacting pathways and precise molecular targets need to be investigated in order to further elucidate the mechanisms underlying epidermal differentiation and carcinogenesis.

## Introduction

The receptor-interacting protein kinase 4 (RIPK4), a member of the RIPK family, was originally identified interacting with protein kinase C (PKC) β and PKCδ by yeast two-hybrid-based screens (1, 2). RIPK4 was first identified as DIK (PKC-delta-interacting protein kinase) in humans. The mouse ortholog of DIK was also found to interact with PKCβ, another PKC isoform, and thus was referred to as PKK (protein kinase C-associated kinase). DIK and PKK have therefore been described as human and mouse RIPK4, respectively (3, 4). RIPK4, containing an N-terminal kinase domain, an intermediate domain, and a unique C-terminal region characterized by the presence of 11 ankyrin repeats, belongs to the serine/threonine kinase family (4). Except for the low-level expression of RIPK4 in the spleen, RIPK4 is widely expressed in a variety of embryonic and mature tissues (e.g., heart, brain, lung, liver, skeletal muscle, kidney, testis) (2, 5). RIPK4 exists in various cytosolic and membrane-associated forms but is not present in the nucleus (2).

Previous studies have indicated that RIPK4 can modulate the actin cytoskeleton and restrict intercellular adhesion (6). Skin-specific loss of RIPK4 results in delayed keratinization, stratum corneum maturation, and aberrantly lipid distribution (7). Therefore, RIPK4 is essential for keratinocyte differentiation and the formation of a normal epidermal barrier (6). In addition, RIPK4 expression is strongly downregulated in keratinocytes early after skin wounds, indicating that RIPK4 may be a regulator of keratinocyte migration during wound healing (8). RIPK4 also plays a critical role in the maintenance of recirculating mature B cells and B cell development *in vivo* (9). Nevertheless, whether RIPK4 influences keratinocyte proliferation remains controversial and deserves additional investigation (3, 8, 10, 11).

## The Role of RIPK4 in Keratinocyte Differentiation

RIPK4 has emerged as a key regulator of keratinocyte differentiation, cutaneous inflammation, and cutaneous wound repair (8, 12). RIPK4-deficient animals display striking abnormality in skin differentiation. The epidermis of RIPK4 knockout mice contains an outermost layer of parakeratotic cells instead of enucleated squamous cells and is thickened with marked hyperplasia of both spinous and granular layers (3). The epidermis-specific RIPK4 knockout mice have a largely normal expression of epidermal differentiation markers (7). By contrast, RIPK4 full deficiency in mice results in aberrant expression patterns of various differentiation markers, such as abnormal expression of keratin 14 in the granular and outermost parakeratotic layer, as well as filaggrin in the spinous and granular layers (3). The observed differences between epidermal-specific RIPK4 knockout mice and RIPK4 full knockout mice may be due to the timing of RIPK4 ablation (7). Furthermore, RIPK4 knockout leads to perinatal lethality in mice, which was most likely due to the suffocation caused by abnormal epidermal differentiation (3). RIPK4 mutations have been linked to Bartsocas-Papas syndrome (BPS) in human, which is typically characterized by aberrant skin, craniofacial and genital development, and early death (13, 14). RIPK4 mutations are also associated with CHAND syndrome, and the acronym summarizes the main features: curly hair, ankyloblepharon (fused eyelids), and nail dysplasia (15). In general, RIPK4 acts as an important regulator of keratinocyte differentiation.

## RIPK4-Related Signaling Pathways

Although RIPK4 plays a critical role in epidermal development and keratinocyte differentiation, the mechanism by which it integrates upstream signals to trigger specific responses remains elusive. Phorbol 12-myristate 13-acetate (PMA), a small-molecule mimic of diacylglycerol, can potentially activate PKCs (16). As previously mentioned, RIPK4 was initially identified as an interaction partner of the PKC family. PKCs could activate RIPK4, which then induced uncontrolled epidermal inflammatory responses (17). Transgenic mice with epidermal overexpressing RIPK4 reacted hypersensitively to PKC (12, 17). This was in accordance with the finding that RIPK4 knockdown in normal keratinocytes hampered the expression of differentiation markers upon PKC activation (18). Collectively, RIPK4 regulates the keratinocyte differentiation and functions via the PKC pathway (18).

In addition, RIPK4 can regulate several other signaling pathways, such as the NF-κB, Wnt/β-catenin, and RAF/MEK/ERK pathways (19–21). The NF-κB pathway is involved in the regulation of diverse functions, including epithelial tissue proliferation, differentiation, inflammation, and immune responses (22). PIPK4 overexpression can cause the dose-dependent activation of NF-κB and Jun N-terminal kinase (JNK) (4). The balanced NF-κB and JNK activation *in vivo* is vital for the regulation of keratinocyte differentiation (23). Inhibition of RIPK4 expression can enhance IκB level in cultured keratinocytes, indicating the reduced activation of NF-κB and the enhancement of keratinocyte differentiation (8). Inhibition of PMA-induced NF-κB activation by a dominant negative mutant of RIPK4 can be reverted by the co-expression of PKC isoform, PKCβI, suggesting that RIPK4 may act as a functional link between PKCβI and NF-κB activation (4, 19). The mechanism by which the PKCβI expression reverts the dominant negative effect of the RIPK4 mutant is still unclear. It is speculated that overexpression of catalytically active PKCβI may compete out the dominant negative RIPK4 for cellular factors necessary for function (19).

Moreover, though the intact kinase properties of RIPK4 are required for NF-κB activation, catalytically inactive RIPK4 mutants can unexpectedly enhance MEKK2- and MEKK3-induced activation of NF-κB. Hence, RIPK4 can activate the NF-κB pathway in both a kinase-dependent and kinase-independent manner (4, 24). Intriguingly, the phenotype of RIPK4-deficient mice in part resembles that of mice lacking IKKα, which implies that these two NF-κB-related kinases may function in a common pathway to regulate epidermal homeostasis and differentiation (25). RIPK4 functions in a PKC-mediated signaling pathway of NF-κB activation that requires IKKα and IKKβ but is independent of Bcl10 and IKKγ, a regulatory subunit of the IκB kinase complex (19).

In addition to activating NF-κB, RIPK4 can also regulate the Wnt signaling pathway. RIPK4 induces the β-catenin accumulation via phosphorylating DVL2, a receptor protein of the Wnt pathway. Phosphorylation of DVL2 at Ser^298^ and Ser^480^ by RIPK4 favors canonical Wnt signaling (20). Except for the essential functions of the Wnt pathway for cell growth, development, and death (26), the Wnt pathway is also related to keratinocyte differentiation (27). The disruption of Wnt signaling can lead to cleft lip/palate (28).

RIPK4 and interferon regulatory factor 6 (IRF6) can also function as a signaling axis downstream of PKC activation to mediate keratinocyte differentiation (18). RIPK4 promotes keratinocyte differentiation, at least in part by inducing IRF6 transactivator function through the phosphorylation of Ser413 and Ser424 in the C-terminal domain of IRF6. RIPK4 can directly phosphorylate and activate IRF6 to induce the expression of key transcriptional regulators of keratinocyte differentiation, including grainyhead-like 3 (GRHL3) and Ovo-like zinc-finger 1 (OVOL1) (18). The ELF3 gene, an ETS family transcription factor, is regulated downstream of GRHL3 in keratinocytes. RIPK4 may regulate the epidermal barrier functions partly through regulating an IRF6-GRHL3-ELF3 transcriptional network (29). In addition to the regulation of keratinocyte differentiation, the multifaceted role of the RIPK4-IRF6 signaling axis in epithelial homeostasis may also extend to the regulation of inflammation. RIPK4 stimulates the expression of specific proinflammatory cytokines (e.g., CCL5 and CXCL11) in keratinocytes through activating IRF6 (30).

A recent study revealed that the RIPK4 kinase domain could directly phosphorylate IRF6 at Ser90. Furthermore, IRF6 regulated the expression of genes that are involved in the lipid metabolism and tight junctions. The above findings provide the insight into how RIPK4 and IRF6 function to ensure the integrity of the epidermis and offer the mechanistic explanation for why genetic defects in the RIPK4-IRF6 axis lead to developmental syndromes (31). Mutations in human IRF6 give rise to Van der Woude syndrome (VWS), a developmental condition, and popliteal pterygium syndrome (PPS), which is a less severe form of BPS (32). To our knowledge, IRF6 is more than a key transcriptional regulator involved in skin differentiation (33); it has also been reported to exhibit tumor suppressor activity in squamous cell carcinoma (SCC) (34). Hence, loss of IRF6 *per se* or an impaired RIPK4-IRF6 signaling axis may also be related to the development of SCC.

## Interactions of RIPK4-Related Signaling Pathways

**FIGURE 1:**
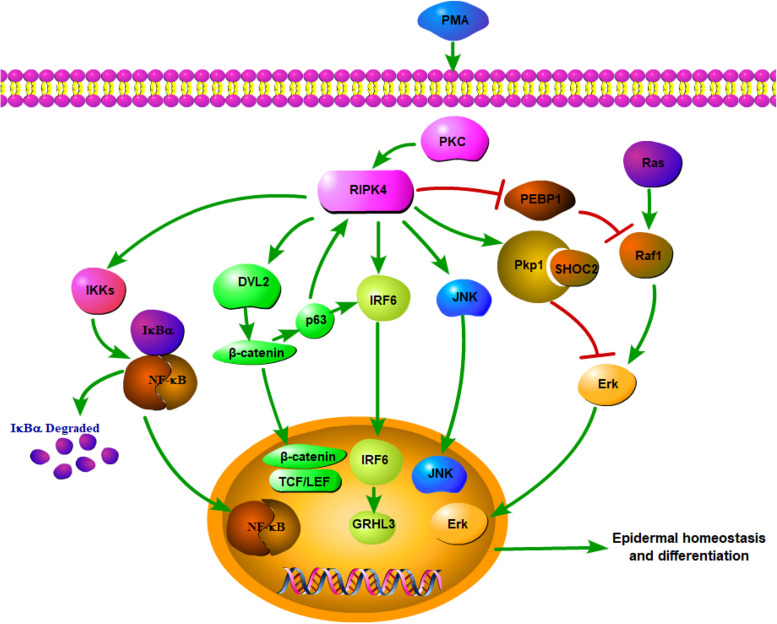
Network of RIPK4 in the multiple signaling pathways for epidermal homeostasis and differentiation.

Overall, RIPK4 functions as a key nodal point for the maintenance of epithelial homeostasis (Figure 1). RIPK4-related biological signaling pathways interact with each other to form a complex network. It is worth noting that both RIPK4 and IRF6 are direct transcriptional targets of the protein p63, which is a master regulator of stratified epithelial development (13, 35). Mice harboring loss-of-function mutations of RIPK4 and IRF6 have close phenotypic similarities (36). Furthermore, Wnt signaling regulates IRF6 expression *in vivo* by transactivating p63 (37). IRF6 may be a novel effector of the Wnt pathway in keratinocytes given that its transactivator function is regulated by RIPK4 (18).

RIPK4 has been proven to be a modulator of the PKC signaling pathway (1). PKCδ activation increases the expression of the transcription factor Kruppel-like factor 4 (KLF4), which upregulates the expression of the differentiation-associated gene, involucrin (IVL) (38). It is worth noting that KLF4 has been identified as an IRF6 target gene (34, 39). Hence, PKCδ may regulate KLF4-mediated IVL transcription via the RIPK4-IRF6 regulatory module.

In addition, the RIPK4 p.Ile121Asn missense mutation, which has been identified in Bartsocas-Papas syndrome, inhibits the kinase activity of RIPK4, thereby abolishing RIPK4-mediated IRF6 and NF-κB activation (14, 18). The mutation also compromises RIPK4-mediated β-catenin stabilization (20). Therefore, the severe manifestation of the RIPK4 p.Ile121Asn missense mutation is likely due to impaired signaling by the RIPK4-IRF6 axis, as well as the NF-κB and Wnt pathways. Another BPS-associated mutation, RIPK4 p.Ser376X nonsense mutation, can impair the IRF6 activation and inhibit its stabilization of β-catenin, while the ability of RIPK4 to activate NF-κB and JNK is unaffected (40). These findings not only suggest molecular bases for how RIPK4 mutations cause epidermal disorders but also provide important mechanistic insights into the regulation of keratinocyte differentiation by a RIPK4-related complex signaling network.

Taken together, RIPK4 plays a vital role in keratinocytes by participating in various signaling transduction pathways. However, the functional significance of RIPK4 in the signaling networks remains unclear. The identification of novel RIPK4-interacting proteins, particularly physiologically relevant substrates, awaits further investigation, which will elucidate the precise role of RIPK4 in a variety of signaling pathways.

## The Role of RIPK4 in Carcinogenesis

As stated above, RIPK4 plays a pivotal role in skin development and keratinocyte differentiation. RIPK4 dysregulation causes the aberrant epidermal differentiation, which may impose a remarkable effect to the occurrence and development of SCC. RIPK4 mutations have been found in several SCCs. The cutaneous SCC (cSCC) data deposited in The Cancer Genome Atlas (TCGA) and the analysis of metastatic cSCC reported a similar high rate of RIPK4 mutagenesis, with mutations clustering within the kinase and ankyrin repeat domains (41, 42). It was strongly implied that the mutations were non-random, thus supporting the hypothesis that RIPK4 is a putative tumor suppressor for cSCC. The RIPK4 mutations have also been identified in human head and neck SCC (HNSCC) through large-scale sequencing, implicating its critical function in squamous epithelial differentiation and carcinogenesis (43). In addition, RIPK4 point mutation was observed in esophageal SCC and RIPK4 was thus considered as the driver gene for this malignancy (44). A significant enrichment of somatic mutations in RIPK4 was also confirmed in human papillomavirus (HPV)-positive oral SCCs (45).

In addition to mutations, RIPK4 is aberrantly expressed in various kinds of cancers, including skin, ovarian, cervical SCCs (10, 20, 46). Compelling evidence highlighted the essential and specific role of RIPK4 as a tumor suppressor in epidermal keratinocytes and RIPK4 deficiency in the skin epidermis greatly impaired skin differentiation and enhanced skin carcinogenesis. RIPK4 expression was decreased in human cSCC in comparison with adjacent skin (11). In a novel RIPK4 conditional knockout mouse model, the loss of keratinocyte RIPK4 promoted SCC formation during chemically induced carcinogenesis (47). RIPK4 could phosphorylate the N-terminal domain of desmosome protein plakophilin-1 (Pkp1), which was essential for RIPK4-mediated epidermal differentiation. The novel RIPK4-Pkp1 signaling axis could then promote SHOC2 binding with Pkp1 (10). This interaction functionally suppressed the Ras/MAP kinase signaling pathway, which played a key role in epidermal homeostasis (48). It was also noteworthy that RIPK4 expression was reduced in tongue SCC and its expression was positively associated with a favorable prognosis. RIPK4 knockdown enhanced migration and invasion capabilities of tongue cancer cells, thus implying that RIPK4 might be a tumor suppressor (49). Similarly, RIPK4 has also been identified as a putative and novel tumor suppressor in human hepatocarcinogenesis. RIPK4 was significantly suppressed in 80% of the hepatocellular carcinoma (HCC) samples, and RIPK4 overexpression resulted in almost complete elimination of anchorage-independent growth in the already transformed human fetal hepatocytes. The role of RIPK4 as a tumor suppressor involved in HCC development might relate to NF-κB signaling (50). The suppression of RIPK4 regulated NF-κB signaling with the increased acquisition of oxidative stress-induced genetic changes and an expansion of pre-malignant subclones (51). Furthermore, bioinformatics analyses of human lung adenocarcinoma samples indicated that poorly differentiated tumors express significantly lower levels of RIPK4, which was associated with poorer overall survival. NF-κB and signal transducer, as well as activator of transcription 3 (STAT3), were two transcription factors often activated in lung adenocarcinoma (52, 53). They also promoted dedifferentiation (54, 55), which was linked to various features of cancer development such as invasion, metastasis, and stemness of cancer cells. Quite strikingly, RIPK4 knockdown leading to lung cancer dedifferentiation was NF-κB independent and the potential of RIPK4 in reducing lung cancer cells metastases was kinase-independent. The loss of RIPK4 enhanced the STAT3 pathway in lung cancer cells and promoted the expression of extracellular matrix (ECM) remodeling genes (56).

On the contrary, mounting evidence has demonstrated the oncogenic role of RIPK4 (20, 21, 46, 57–59). RIPK4 knockdown in A2780 and COV434 ovarian cancer cells could inhibit β-catenin accumulation and RIPK4 overexpression could promote ovarian cancer in a xenograft tumor model (20). In addition, RIPK4 was significantly upregulated in osteosarcoma and RIPK4 knockdown suppressed EMT by inactivating Wnt/β-catenin signaling (60). RIPK4 promoted Wnt signaling, implying that RIPK4 overexpression might be involved in the development of certain tumor types. However, RIPK4 might only act as an oncogene in Wnt-dependent tumors, since RIPK4 knockdown had no effect on Wnt3a-induced β-catenin accumulation in pancreatic PANC1 cells, kidney 786-O cells, and breast HCC38 or HS578T cells (20). Additionally, RIPK4 did not enhance the transcription activity of β-catenin in cervical SCC cells. A pro-metastasis function of RIPK4 was observed in the progression of cervical SCC, since RIPK4 promoted the epithelial–mesenchymal transition (EMT) process (46).

Moreover, RIPK4 exerts an oncogenic role through activating NF-κB signaling, which is involved in the pathogenesis of some malignant diseases. Meylan et al. reported a dose-dependent activation of NF-κB in 293T cells by overexpression of RIPK4 (4). RIPK4 was required for the survival of human diffuse large B-cell lymphoma (DLBCL) cells primarily through controlling NF-κB activation induced by the B cell-activating factor of the tumor necrosis factor family (BAFF). RIPK4 inactivation inhibited NF-κB activity, impaired the survival of DLBCL cells, and sensitized DLBCL cells to the treatment with chemotherapeutic agents (57). Consistent with previous studies in bladder urothelial carcinoma, the oncogenic activity of RIPK4 depended on the activation of NF-κB, leading to increased vascular endothelial growth factor A (VEGF-A) levels, which ultimately mediated the RIPK4-induced EMT and promoted bladder urothelial carcinoma cell aggressiveness (58). RIPK4 overexpression promoted the growth of nasopharyngeal carcinoma (NPC) cells. RIPK4 could activate NF-κB signaling by enhancing the interaction between IKKα and IKKβ and improving the stability of the IKK complex (59). Hence, RIPK4 might regulate NF-κB signaling at multiple levels, with differing molecular mechanisms in different cell types.

Compared to normal pancreatic tissues, RIPK4 was upregulated in the subgroup of pancreatic cancer with a high metastatic potential (21). Phosphatidylethanolamine-binding protein 1 (PEBP1) was a physiological endogenous inhibitor of the mitogen-activated protein kinase (MAPK) pathway; the effect of PEBP1 on the MAPK pathway could be regulated by PKC (61, 62). Notably, as a downstream signaling molecule of PKCδ, RIPK4 overexpression promoted pancreatic cancer cell migration and invasion via the proteasome-mediated PEBP1 degradation-induced activation of the RAF1/MEK/ERK signaling pathway (21). The activation loop of RIPK4 contains a Ser-*X*-*X*-*X*-Ser motif, implying that RIPK4 is related to MAP kinase kinases and it may be also activated *in vivo* by a MAP kinase kinase kinase. PKCδ has been found to participate in MAPK signaling (63). Therefore, it is reasonable to assume that RIPK4, as a protein kinase that interacts with PKCδ, may serve as an intermediary in PKCδ and MAPK pathway. Further studies are needed to identify the MAP kinase kinase kinase required for the activation of RIPK4 (2).

## Discussion

RIPK4 is essential for skin development during embryogenesis and normal skin tissue homeostasis in adult animals through regulating epidermal differentiation. To date, the critical role of RIPK4 in the pathogenesis of malignant diseases has not been extensively investigated. The current controversy concerning the potential role of RIPK4 in carcinogenesis indicates that RIPK4 may have a context-specific function in carcinogenesis and/or different signal transduction mechanisms in different tumor types. Although the previous studies have not fully elucidated the mechanism by which RIPK4 promotes carcinogenesis, the observations of the association between RIPK4 and carcinogenesis may have prognostic and therapeutic implications.

As mentioned above, RIPK4 expression is positively associated with favorable prognosis in tongue SCC and lung adenocarcinoma (49, 56). By contrast, increased RIPK4 expression predicts a poor prognosis in cervical SCC, pancreatic cancer, bladder cancer, and osteosarcoma (21, 46, 58, 60). RIPK4 is a novel and independent prognostic factor, which may be used to identify patients at risk and guide individualized treatment.

Furthermore, targeting the RIPK4 pathway may be a promising therapeutic strategy for the treatment of patients with a specific malignancy. Natural halloysite nanotube (HNT)–assisted delivery of an active small interfering RNA targeting RIPK4 efficiently inhibited RIPK4 expression *in vivo* and suppressed bladder cancer tumorigenesis and progression with no adverse effects. Further analysis indicated that the NF-κB signaling pathway might be involved as the downstream pathway for the regulation of proliferation and progression in bladder cancer (64). RIPK4 is a critical activator of NF-κB signaling, which contributes to cancer development and progression, as well as to the resistance of cancer cells to chemoradiotherapy (65). Thus, targeting RIPK4 may be a viable approach for cancer therapy.

So far, there is little known about the physiological upstream signals that regulate RIPK4. However, understanding the activation mechanism of RIPK4 gives valuable information about how RIPK4 functions at the biochemical level and how it controls epidermal differentiation and carcinogenesis. Constitutively activated RIPK4 by PKC can be degraded by the SCF^β–*TrCP*^-mediated proteasomal degradation pathway to maintain cortical actin organization in cultured keratinocytes (17). β-TrCP, which regulates multiple signaling pathways controlling cell growth, survival, death, and differentiation, is found to be involved in the process of carcinogenesis (66, 67). Consistent with RIPK4’s dual oncogenic and tumor-suppressive effects depending on the cellular context, β-TrCP also exerts dual functions in tumor development and progression (67).

Expression level, subcellular localization, and tissue localization of RIPK4 in human cancers should be further clarified to improve our understanding of the biological bases of cancer progression. Additional functional experiments are needed to identify the novel RIPK4-interacting partners and elucidate the precise molecular mechanisms of RIPK4 in different kinds of cancer. There is also great need to uncover the other signaling networks that are regulated by RIPK4 in both normal skin differentiation and carcinogenesis.

## Author Contributions

JX was involved in writing the article. QW was involved in revising the article. ZH was involved in editing the article and supervising the work. All authors contributed to the article and approved the submitted version.

## Conflict of Interest

The authors declare that the research was conducted in the absence of any commercial or financial relationships that could be construed as a potential conflict of interest.
